# PIWIL4: from biological characteristics to disease pathogenesis and clinical applications

**DOI:** 10.3389/fonc.2026.1893773

**Published:** 2026-09-14

**Authors:** Qi Deng, Yi Liu, Yang Qu, YanQiong Yu, YuMing Liu, QiBing Yan, YongKang Wu

**Affiliations:** 1 West China Hospital, Sichuan University Jintang Hospital. Jintang First People’s Hospital, Chengdu, China; 2 West China Hospital, Sichuan University, Chengdu, China

**Keywords:** PIWIL4, PIWI/piRNA pathway, context-dependent function, functional duality, epigenetic regulation, therapeutic targets

## Abstract

PIWIL4 is a core component of the PIWI/piRNA pathway. Its roles in germ cell development, transposon silencing, and genome stability maintenance are well established. Recent studies have shown that PIWIL4 is abnormally expressed in many cancers. There, it helps regulate key malignant processes—including cell cycle control, apoptosis, epithelial-mesenchymal transition, stemness maintenance, and metabolic reprogramming. However, its effects vary widely, it can promote or suppress tumours depending on the cancer type, and even across subtypes of the same cancer. This striking functional duality is highly context-dependent. This review systematically covers the molecular structure, classical functions, and complex regulatory networks of PIWIL4 in both cancerous and non-cancerous conditions. We focus on the molecular underpinnings of this duality—specifically, piRNA-guided targeting, differences in protein interaction networks, and the influence of cellular context. We also assess its promise and hurdles as a diagnostic biomarker and therapeutic target. A deeper understanding of these context-dependent mechanisms should inform the design of targeted interventions in precision oncology.

## 1 Introduction

*PIWIL4* (Piwi-like RNA-mediated gene silencing 4) is a member of the PIWI subfamily within the Argonaute protein family. Its gene resides on human chromosome 11q12 (1). The protein was first discovered in Drosophila germline stem cells, where it serves as a key regulator of self-renewal. The mammalian PIWI family has four members (PIWIL1–4). Among them, PIWIL4 stands out because of its distinctive molecular features. Unlike PIWIL1 and PIWIL2, PIWIL4 has no endonuclease activity. Instead, it plays a central role in the piRNA amplification cycle, guiding secondary piRNAs to nuclear target loci and initiating epigenetic silencing (2–5). In its classical role, PIWIL4 forms an effector complex with piRNA during spermatogenesis. This complex recognizes newly synthesized transposon transcripts and recruits DNA methyltransferases (DNMTs) and histone methyltransferases. These enzymes deposit Deoxyribonucleic Acid (DNA) methylation and Histone H3 lysine 9 trimethylation(H3K9me3) marks at transposon loci, driving heterochromatin formation and transcriptional silencing (6–8). This mechanism is critical for preserving germline genome integrity. When PIWIL4 function fails or piRNA binding is impaired, transposons like Long Interspersed Nuclear Element-1(LINE-1)become de-repressed. This triggers DNA damage and disrupts spermatogenesis (9–15). PIWIL4 localizes to both the nucleus and the cytoplasm (16). In the cytoplasm, it resides in piP-bodies (a subtype of nuage structures), where it participates in piRNA processing and transfer (17). In the nucleus, it binds newly transcribed transposon transcripts to mediate epigenetic silencing (18, 19). Over the past few years, research on PIWIL4 has moved beyond germ cells into somatic cells and tumour biology. PIWIL4 is present in many somatic tissues, where it takes on non-classical roles—regulating insulin secretion in pancreatic β-cells (20) and maintaining retinal epithelial homeostasis (21, 22). More notably, PIWIL4 is overexpressed in a range of malignancies, where it drives tumour initiation and progression through diverse mechanisms. In breast cancer, PIWIL4 complexes with PIWI-interacting RNA-31115 (piR-31115) to block Heat Shock Protein 90 Alpha Family Class A Member 1 (HSP90AA1) degradation. This in turn boosts the migration of triple-negative breast cancer cells (23, 24). In retinoblastoma, the PIWIL4/piRNA complex may modulate *Orthodenticle Homeobox 2 (OTX2)* cis-regulatory elements through epigenetic changes, helping preserve the stem-like traits of cancer stem cells (25, 26). In acute myeloid leukaemia (AML), PIWIL4 takes on a non-canonical role: it acts as an R-loop helicase that clears accumulated R-loops, thereby preserving genomic stability in leukaemia stem cells (27, 28). PIWIL4 is also overexpressed in several solid tumours, where it appears to promote cancer progression—including gastric cancer (29, 30), cervical cancer (31), hepatocellular carcinoma (HCC) (32–36), colorectal cancer (CRC) (37, 38), and pancreatic cancer (39, 40). Notably, PIWIL4 function varies considerably across tumour types—and even across subtypes of the same tumour. In some cancers—including fibrosarcoma (41), renal cell carcinoma (42, 43), and soft tissue sarcoma (1)—high PIWIL4 expression correlates with better prognosis, hinting at a tumour-suppressive role. This “context-dependent” duality—the same molecule driving or suppressing tumours depending on the tissue environment—is PIWIL4’s most striking feature. It also poses a central challenge for designing PIWIL4-targeted therapies. Here we review the molecular structure and classical functions of PIWIL4, along with its diverse roles in cancer and non-cancer diseases. We then examine the molecular basis of its functional duality and its clinical potential, aiming to provide a framework for developing PIWIL4 into a useful diagnostic marker and therapeutic target.

## 2 The molecular structure, classical functions and subcellular localisation of PIWIL4

The *PIWIL4* protein is 852 amino acids long. Its structure has three main parts: an N-terminal region, a central PAZ (Piwi/Argonaute/Zwille) domain, and a C-terminal Piwi domain (2–4). The PAZ domain recognizes and binds the 3′ end of piRNAs (3). The PIWI domain is structurally similar to RNase H, but PIWIL4 itself has no endonuclease activity. This sets it apart from PIWIL1 and PIWIL2, both of which have “slicer” function and take part in the piRNA “ping-pong” amplification cycle. As a “slicer-incompetent” PIWI protein, PIWIL4 mainly directs secondary piRNAs to nuclear targets and initiates epigenetic silencing (5). In its classical role during spermatogenesis, PIWIL4 forms an effector complex with piRNAs. This complex recognizes newly transcribed transposon transcripts and recruits DNMTs and histone methyltransferases. These enzymes then induce DNA methylation and H3K9me3 at transposon loci, promoting heterochromatin formation and transcriptional silencing (6–8). This mechanism is essential for germline genome integrity. When PIWIL4 function fails or piRNA binding is impaired, transposons like LINE-1 become derepressed. The result is DNA damage and spermatogenic defects (9–15). PIWIL4 is found in both the nucleus and the cytoplasm (16). In the cytoplasm, it resides in piP-bodies—a subtype of nuage structures—where piRNA processing and transfer between PIWIL2 and PIWIL4 take place (17). In the nucleus, it binds nascent transposon transcripts to mediate epigenetic silencing (18, 19). The nuclear-cytoplasmic shuttling of PIWIL4 is developmentally regulated. During germline epigenetic reprogramming, for instance, PIWIL4 moves dynamically from the nucleus to the cytoplasm. **Figure 1** illustrates the domain architecture of PIWIL4, the classical piRNA pathway, and its subcellular distribution.

**Figure 1:**
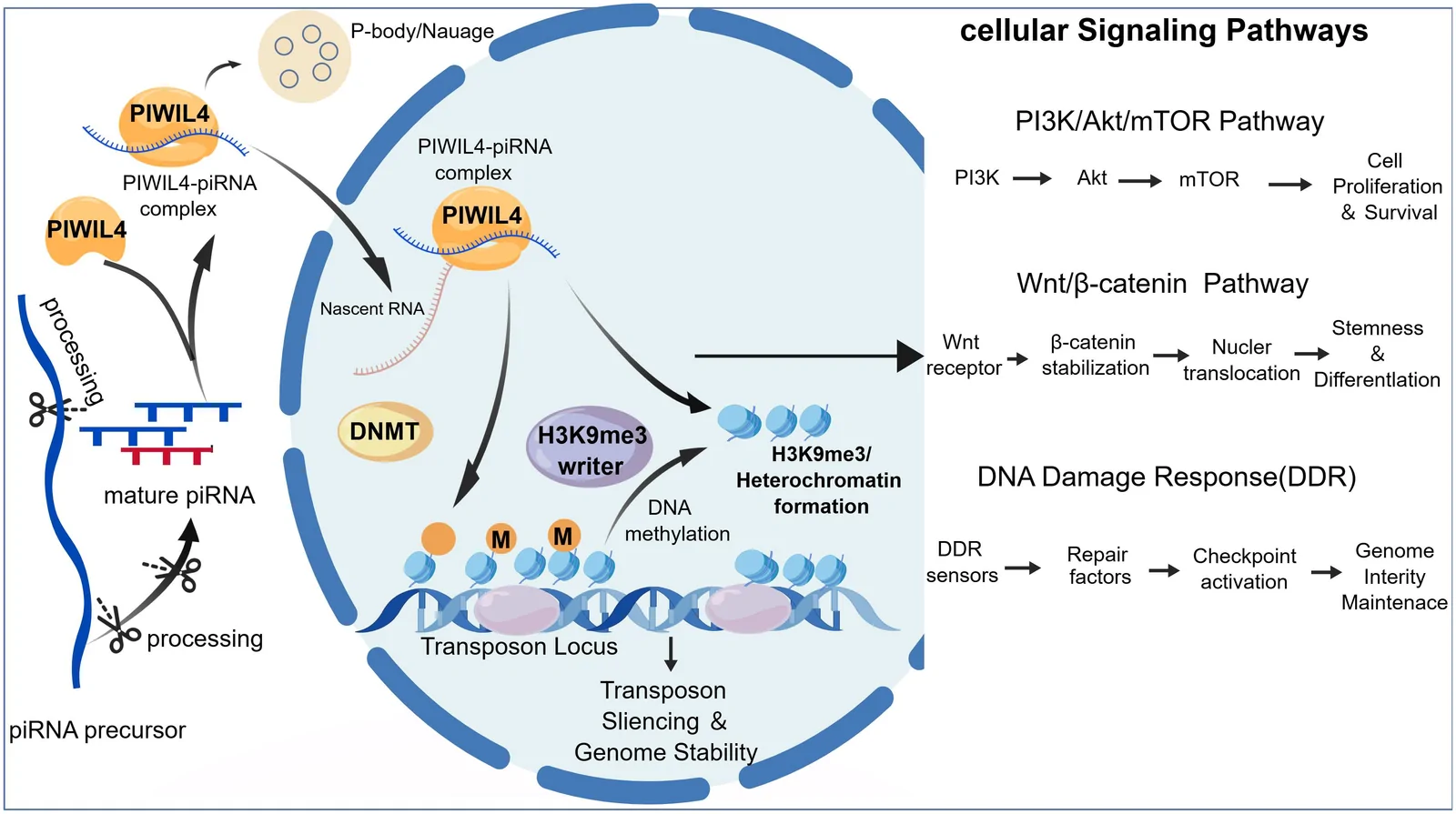
Domain architecture of PIWIL4, the canonical piRNA pathway, and its subcellular distribution. PIWIL4 mainly acts through the PIWI/piRNA pathway. In this pathway, piRNA precursors are transcribed from genomic piRNA clusters and then moved to the cytoplasm. There, they are processed into mature piRNAs—26 to 31 nucleotides long—with a 2′-O-methyl modification at their 3′ end. These mature piRNAs then load onto PIWIL4, forming the PIWIL4/piRNA effector complex. This complex is then transported into the nucleus. Inside the nucleus, the complex uses sequence complementarity between the piRNA and its target nascent transcript to recognize matching transposon mRNAs. It then recruits epigenetic enzymes—including DNMTs and histone methyltransferases that deposit H3K9me3. These enzymes trigger DNA methylation, heterochromatin formation, and transcriptional silencing at the transposon locus, thereby preserving genomic stability. Beyond the nucleus, PIWIL4 and its associated complexes also reside in cytoplasmic processing bodies (P-bodies) and nuage structures. There, they take part in post-transcriptional regulation—including mRNA degradation and translation inhibition. PIWIL4 also engages in crosstalk among several cellular signalling pathways—PI3K/Akt/mTOR (proliferation), Wnt/β-catenin (stemness maintenance), and the DNA damage response (genomic integrity). This broadens its regulatory influence beyond piRNA-mediated silencing. PIWIL4’s presence in both the nucleus and the cytoplasm, along with its interactions with various partners and signalling networks, underpins its functional diversity and context-dependent behaviour in both health and disease. Graphical legends: Solid arrows represent molecular assembly, nuclear translocation and positive regulatory activation; scissor icons indicate RNA cleavage and processing.

## 3 The mechanistic basis of PIWIL4 as a “context-dependent” regulatory factor

### 3.1 The mechanistic pathways through which PIWIL4 acts as an oncogene

#### 3.1.1 Cell cycle regulation and proliferation drivers

PIWIL4 drives abnormal cell proliferation in many cancers by disrupting key cell cycle checkpoints. In gastric cancer, PIWIL4 is markedly overexpressed in tumour tissues. Its expression levels correlate positively with T stage, lymph node metastasis, and cTNM stage. Knocking down *PIWIL4* triggers G_2_/M arrest and suppresses cell proliferation (29, 44). Wang and their colleagues further confirmed by immunohistochemistry (IHC) that PIWIL4 is highly expressed in gastric cancer tissues and correlates with poor prognosis (29, 30). Of note, this effect is not confined to gastric cancer. In fibrosarcoma, PIWIL4 also triggers G_2_/M arrest, but with a very different outcome (41) (discussed further in the Fibrosarcoma section). This suggests that cellular context dictates whether G_2_/M arrest leads to growth inhibition or apoptosis. In hormone-dependent breast cancer, the oestrogen/ERα pathway regulates PIWIL4, which in turn boosts cell proliferation, migration, and invasion by modulating Estrogen Receptor (ER) target genes and EMT(epithelial-mesenchymal transition)-related molecules (45). In triple-negative breast cancer, however, a hormone-independent mechanism comes into play: PIWIL4 promotes migration via its interactions with piR-31115 and HSP90AA1 (23). This difference underscores the functional heterogeneity of PIWIL4 across breast cancer subtypes.

#### 3.1.2 Apoptosis inhibition and survival signalling: inactivation of the p53 pathway and mitochondrial apoptosis resistance

Apoptosis evasion is a hallmark of cancer. In several cancers, PIWIL4 helps tumour cells resist death signals by disrupting the p53 tumour suppressor network and the mitochondrial apoptosis pathway. This promotes cell survival and fuels disease progression.

##### 3.1.2.1 PIWIL4 targets the p14ARF/p53 pathway in cervical cancer

In cervical cancer, PIWIL4 promotes cell survival mainly by epigenetically regulating the *CDKN2A* locus. This locus encodes two important tumour suppressors: p16INK4a (which inhibits cell cycle progression) and p14ARF (which activates the p53 pathway) (46, 47). Ozenne et al. found that PIWIL4 is markedly overexpressed in cervical cancer tissues and in HeLa and C33A cells (48). Its growth-promoting effect comes from suppressing apoptosis, not from disrupting the cell cycle (31). Mechanistically, p14ARF normally binds to and stabilizes p53. PIWIL4, however, reduces p14ARF levels by interfering with MDM2-mediated ubiquitination and degradation of p53 (48). This drop in p14ARF inactivates the p53 pathway, weakening the ability of cancer cells to trigger apoptosis under stress. At the same time, PIWIL4 also lowers p53 expression itself (31). This creates a two-pronged inhibitory effect that blocks the upstream signals of the mitochondrial apoptosis pathway. This mechanism resembles the methylation-driven silencing of CDKN2A seen in Malignant Melanoma (49, 50). It suggests that PIWIL4 may serve as a broad epigenetic regulator, targeting key tumour suppressor loci to release the p53 pathway in multiple cancers.

##### 3.1.2.2 PIWIL4 regulates the mitochondrial pathway in Leydig cell tumours

Leydig cell tumours are rare mesenchymal tumours. For cases that have become malignant, no effective treatments currently exist. Wang et al. established a mouse model and found that most evidence for PIWIL4’s function in this context comes from reverse pharmacology studies. Their work showed that liquiritigenin induces apoptosis in mouse MLTC-1 Leydig tumour cells. Key molecular changes include downregulation of PIWIL4, loss of mitochondrial membrane potential, and sequential activation of caspase-9 and caspase-3—all hallmarks of intrinsic (mitochondrial) apoptosis (51). Treatment with icariin produced similar effects, reinforcing the idea that PIWIL4 has an anti-apoptotic role in this model (51). Notably, in Leydig cell tumours, apoptosis triggered by PIWIL4 downregulation does not rely on classical p53 transcriptional activation—unlike in cervical cancer. Instead, it may involve adjusting the Bcl-2 family protein balance or directly altering mitochondrial outer membrane permeability (MOMP). This difference underscores the context-dependent nature of PIWIL4’s anti-apoptotic mechanism. In cells with an intact p53 pathway, PIWIL4 may work through the p14ARF/p53 axis. In cells with mutant or nonfunctional p53, it may act directly on the mitochondrial apoptosis machinery (51).

#### 3.1.3 PIWIL4 drives EMT and metastasis through tissue-specific regulatory networks

Metastasis is the main cause of cancer-related deaths. The EMT is the key cellular process that sets off the metastatic cascade (39). PIWIL4 participates in EMT regulation across many cancers. However, its effects are highly tissue-specific. It engages classical EMT transcription factors, growth factor signalling pathways, and non-coding RNA networks—depending on the cellular context (39, 52, 53) (**Table 1**).

**Table 1:** Shared mechanisms and tissue-specific differences in PIWIL4’s regulation of EMT and metastasis.

Disease	Pancreatic cancer	Breast cancer (ER+)	Breast cancer (TNBC)	Lung cancer
Upstream activation signal	Unknown (possibly piRNA-mediated)	Estrogen/ERα	piR-31115/HSP90AA1	PLIC11/YY1 Axis
Core Mechanism of Action	Direct regulation of EMT transcription factors (Snail/Slug/Vimentin)	Regulation of EMT molecules and ER target genes	Stabilisation of HSP90AA1 → multi-pathway activation	Transcriptome reprogramming (including EMT-related)
Functional consequences	Metastasis↑, chemotherapy resistance↑	Metastasis↑, Invasion↑	Metastasis↑	Proliferation↑, invasion↑, metastasis↑
Prognostic significance	Associated with a favourable prognosis	Not specified	Not specified	Controversial (low expression is associated with a poor prognosis)

↑, increased/enhanced biological phenotype. →, indicates causal or sequential biological progression.

##### 3.1.3.1 PIWIL3 and PIWIL4 co-regulate the EMT transcriptional programme in pancreatic cancer

In pancreatic ductal adenocarcinoma (PDAC), PIWIL3 and PIWIL4 work together to regulate EMT through a relatively direct mechanism. Li et al. analysed pancreatic cancer tissues and cell lines (ATCC CRL-2555). They found that co-silencing PIWIL3 and PIWIL4 markedly reduced mesenchymal markers (vimentin, Snail, and Slug) while raising epithelial markers (E-cadherin). This shift reversed the mesenchymal phenotype of the tumour cells (39).

Beyond these molecular changes, the functional phenotype also shifts markedly. Silencing PIWIL3/PIWIL4 does more than reduce tumour cell migration and invasion—it also reverses their dedifferentiated state and makes them more sensitive to drugs like gemcitabine (39). This points to two important conclusions. First, PIWIL4-driven EMT is a core mechanism behind the malignant behaviour of pancreatic cancer. Second, targeting the PIWIL4-EMT axis could both curb metastasis and boost chemosensitivity—offering a new strategy for this hard-to-treat cancer. Gonzalez-Gonzalez et al. further uncovered the prognostic significance of PIWIL4 in pancreatic cancer. Higher PIWIL4 expression correlates with better outcomes and appears to regulate specific downstream piRNAs (40). This raises the possibility that the PIWIL4/piRNA axis fine-tunes EMT-related gene expression at the post-transcriptional level. The same study showed that a liquid biopsy panel using PIWIL4-associated piRNAs improves Carbohydrate Antigen 19-9 (CA19-9) sensitivity for detecting pancreatic cancer (40). This directly connects EMT regulation to clinical practice.

##### 3.1.3.2 Growth factor pathways drive PIWIL4-mediated EMT in breast cancer subtypes

In breast cancer, PIWIL4’s regulation of EMT depends heavily on the molecular subtype. This makes breast cancer a prime example of PIWIL4’s context-dependent behaviour. In hormone receptor-positive (ER+) invasive breast cancer, the oestrogen/ERα pathway directly controls PIWIL4 transcription. Once activated, ERα boosts PIWIL4 expression. PIWIL4 then enhances cell proliferation, migration, and invasion by modulating ER target genes and EMT markers (E-cadherin and vimentin) (45). This places PIWIL4 at the intersection of hormone signalling and metastasis. It suggests that in ER+ breast cancer, PIWIL4 may drive resistance to endocrine therapy and promote late-stage spread.

In triple-negative breast cancer (TNBC), however, hormone-independent mechanisms take over. Wang et al. reported that PIWIL4 is highly expressed in MDA-MB-231 cells (a TNBC line). Knocking it down markedly reduces cell migration and triggers apoptosis (52). Mechanistically, this involves the TGF-β/MAPK/ERK and FGF pathways, along with changes in MHC class II expression (52). These are all known drivers of EMT and metastasis.

Recent work has shed more light on how PIWIL4 is fine-tuned in TNBC. Du et al. found that PIWIL4 complexes with piR-31115. This complex binds HSP90AA1 and prevents its degradation (23). HSP90AA1 is a key chaperone for several EMT-related pathways, including TGF-β receptor, AKT, and STAT3. When stabilized by PIWIL4, it boosts tumour cell migration. This finding aligns well with the prognostic role of the PIWI/piRNA pathway in breast cancer reported by Krishnan et al. (24). Together, these studies reveal a unique mechanism: PIWIL4 regulates EMT through a non-coding RNA–chaperone axis.

Taken together, these findings show that in breast cancer, the PIWIL4-EMT network responds to different upstream signals—hormones in some subtypes, non-coding RNAs in others. Yet it ultimately converges on the same downstream targets: the EMT transcriptional programme and cytoskeletal dynamics (**Figure 2**).

**Figure 2:**
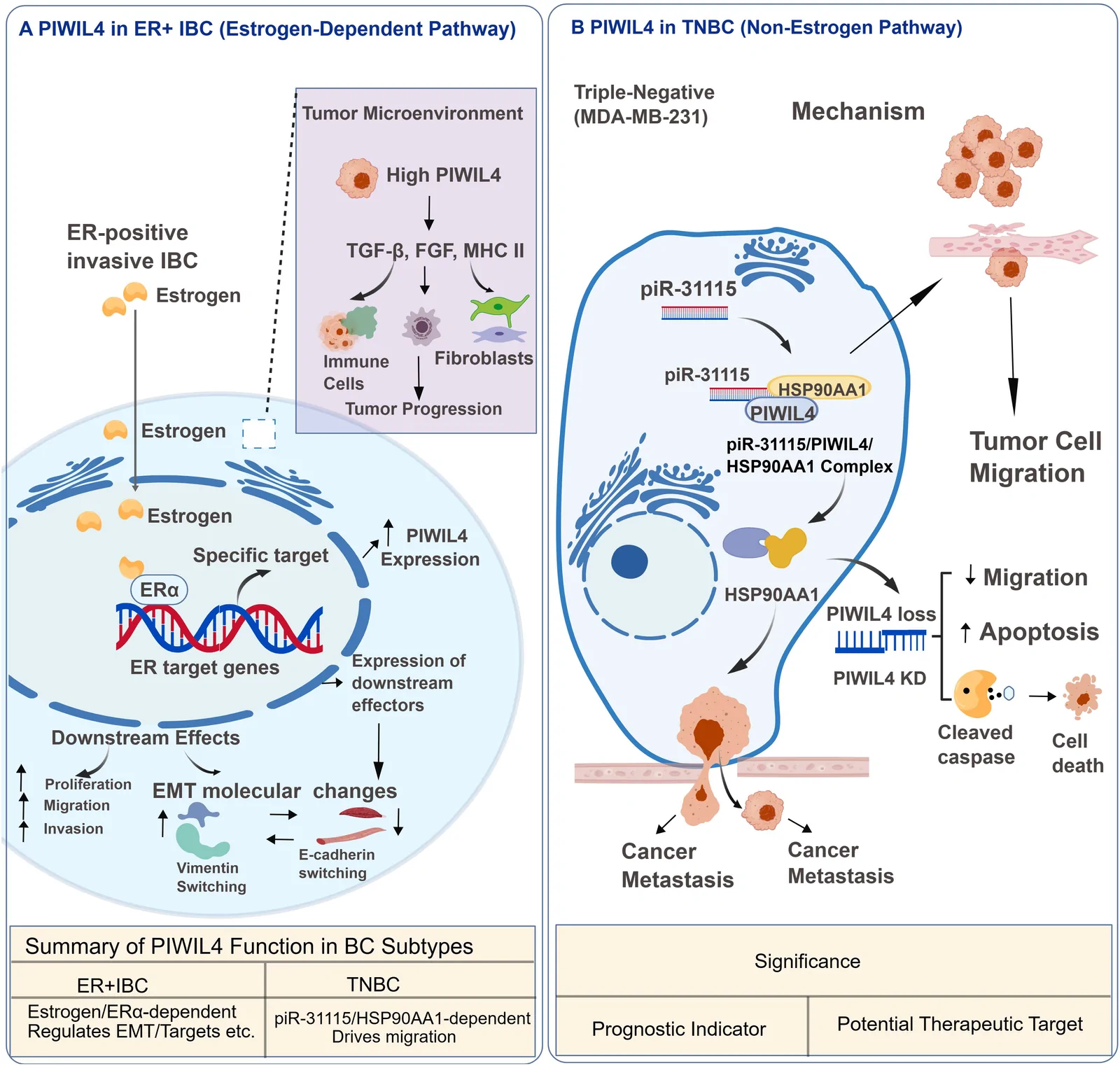
PIWIL4 regulatory networks differ between the two main breast cancer subtypes. **(A)** Oestrogen-dependent pathway (ER-positive invasive breast cancer). Oestrogen and tumour microenvironment signals drive high PIWIL4 expression. This upregulates downstream effectors—TGF-β, FGF, and MHC class II molecules—which together induce EMT. The result is vimentin upregulation and E-cadherin downregulation, ultimately fuelling metastasis. In this context, PIWIL4 serves as a transcriptional co-regulator linked to ERα, bridging hormone signalling and metastatic reprogramming. **(B)** Non-oestrogen-dependent pathway (triple-negative breast cancer). Here, piR-31115 regulates PIWIL4. They form a ternary complex with HSP90AA1 or HSP90AA2. This complex stabilizes PIWIL4 and boosts its pro-migratory activity. Knocking down PIWIL4 suppresses migration, invasion, proliferation, and anti-apoptotic capacity in TNBC cells. Overexpression produces the opposite effect. Because this pathway operates independently of oestrogen, it underscores the functional plasticity of PIWIL4 across breast cancer subtypes. Graphical conventions: Solid arrows represent transcriptional activation or positive functional promotion; bar-ended lines indicate inhibitory regulation; dashed lines denote indirect paracrine effects; upward/downward arrows mean upregulation/downregulation of biological phenotypes.

##### 3.1.3.3 A transcriptional axis drives PIWIL4-mediated metastasis in lung cancer

In lung cancer—mainly non-small cell lung cancer (NSCLC)—PIWIL4 promotes metastasis through a distinct transcriptional axis: PLIC11/YY1/PIWIL4. Zhu et al. systematically mapped this pathway. PLIC11 (a protein not yet fully characterized) upregulates YY1 expression and boosts its transcriptional activity. Activated YY1 then binds the PIWIL4 promoter, driving PIWIL4 transcription (53). As a core PIWI/piRNA effector, PIWIL4 then drives lung cancer cell proliferation, invasion, and metastasis—either by regulating target genes or through epigenetic changes (53). Unlike its more direct regulation of EMT markers in pancreatic and breast cancers, PIWIL4’s downstream effects in lung cancer may involve broader transcriptomic reprogramming. This includes coordinated changes in the cytoskeleton, matrix-degrading enzymes (such as MMPs), and adhesion molecules. The link between PIWIL4 expression and prognosis in lung cancer remains debated. Navarro et al. found that PIWIL4 is markedly downregulated in tumour tissues relative to normal tissues. Patients with low PIWIL4 expression had shorter recurrence-free and overall survival (54). Li et al. confirmed this using several risk models (55). In contrast, functional work by Zhu et al. supports a clear oncogenic, pro-metastatic role for PIWIL4 (53). This apparent contradiction may reflect subtype- or stage-specific differences. PIWIL4 might play a protective role early on, but become hijacked by aberrantly activated transcriptional axes (like PLIC11/YY1) as the tumour advances—turning into a driver of metastasis. This idea, however, needs further testing.

#### 3.1.4 PIWIL4 maintains cancer stem cell properties through epigenetic reprogramming

Cancer stem cells (CSCs) are a subset of tumour cells that can self-renew and differentiate into multiple lineages. They are thought to drive tumour initiation, relapse, metastasis, and therapy resistance (56). Epigenetic reprogramming—including DNA methylation, histone modifications, and chromatin remodelling—plays a key role in preserving CSC stemness (56, 57). As a PIWI/piRNA effector, PIWIL4 helps maintain CSC stemness across various tumours via piRNA-guided chromatin regulation. At its core, the PIWIL4/piRNA complex targets key transcription factor loci and modulates their expression through epigenetic changes. This locks CSCs into an undifferentiated state.

##### 3.1.4.1 PIWIL4 maintains stemness and drives chemoresistance in ovarian cancer stem cells

Ovarian cancer is highly heterogeneous and quickly develops chemotherapy resistance. These traits are closely tied to the presence of cancer stem cells (CSCs). Sohn and Oh systematically profiled PIWI family proteins (PIWIL1–4) and their associated piRNAs in OCSCs (58). They found that high PIWIL3/4 expression is a core feature of OCSCs and correlates strongly with platinum-based chemotherapy response. Specifically, patients who did not respond to cisplatin within 6 months of recurrence-free survival (RFS) had much higher PIWIL4 levels than responders (58). This suggests PIWIL4 could serve as a predictive biomarker for RFS in ovarian cancer. It also points to a mechanism: PIWIL4 may drive chemoresistance by preserving CSC stemness—since CSCs are inherently resistant to standard chemotherapy. Mechanistically, the PIWI/piRNA pathway silences tumour suppressor genes in OCSCs through epigenetic changes, preserving their self-renewal and stemness (58). Specifically, PIWIL4 may work with its bound piRNAs to target CpG islands or regulatory regions at specific loci. This recruits DNMTs or histone modifiers, leading to transcriptional silencing. Meanwhile, some downregulated piRNAs may affect OCSC traits through Golgi-related processes (58). This hints that dynamic shifts in the piRNA landscape could influence the transition between stem-like and differentiated states. Chen et al. also reported that PIWIL1–4 are markedly overexpressed in both primary and metastatic ovarian tumours—enough to distinguish cancerous from healthy tissue (59). Although this study did not clarify the downstream pathways, it supports a role for PIWI proteins—especially PIWIL4—in ovarian cancer invasion and metastasis. This function likely stems from maintaining the metastatic competence of CSCs.

##### 3.1.4.2 The PIWIL4/piRNA-OTX2 axis regulates stemness in retinoblastoma—a comprehensive epigenetic model

Retinoblastoma (RB) is the most common eye cancer in infants and young children. How its cancer stem cells (CSCs) maintain stemness is a central question in the field. Among all cancers, RB offers the most systematic and detailed view of PIWIL4-mediated epigenetic regulation. It provides a comprehensive model for understanding how PIWIL4 controls stemness. Sivagurunathan et al. first reported that PIWIL4 (called HIWI2 in their study) is markedly upregulated in RB cells. Knocking it out disrupts stemness-related factor expression (25). They also found that PIWIL4 may functionally interact with OTX2—a key transcription factor that maintains the undifferentiated state and stemness of RB cells (25). Roy and Chidambaram then built on this to propose a comprehensive model for the PIWIL/piRNA–OTX2 axis (26). In this model, the PIWIL/piRNA complex recognizes OTX2 cis-regulatory modules (CRMs) and recruits epigenetic modifiers—DNMTs and histone methyltransferases/demethylases—to these regions. This dynamically adjusts chromatin accessibility and transcription factor binding at the OTX2 locus. In RBCSCs, the PIWIL4/piRNA complex keeps the OTX2 CRM in an open chromatin state—possibly by removing repressive marks like H3K27me3 or maintaining active marks like H3K4me3. This ensures sustained OTX2 expression. OTX2 then acts as a lineage-survival factor, promoting CSC self-renewal and proliferation while blocking neuronal differentiation (26). This mechanism is significant because it connects three levels: epigenetic modifications, transcription factor interactions, and cellular differentiation fate. Interfering with PIWIL4–piRNA binding, blocking PIWIL4’s epigenetic control of OTX2 CRMs, or directly inhibiting OTX2 activity—any of these strategies could disrupt stemness pathways and oncogenic signalling, thereby slowing RB progression and recurrence (26). This model offers not only new therapeutic avenues for RB but also a general framework for other cancers where transcription factors are epigenetically regulated by the PIWI/piRNA pathway (25).

##### 3.1.4.3 The LINC00857/PIWIL4 axis remodels the immune microenvironment in colorectal cancer

In CRC, PIWIL4 influences stemness and the tumour microenvironment through a long non-coding RNA (lncRNA)-mediated pathway (37, 38, 60). Ren et al. showed that *LINC00857*—acting as either a competitive endogenous RNA (ceRNA) or a transcriptional regulator—markedly upregulates PIWIL4. The two are positively correlated in expression (38). Functionally, this axis participates in multiple immune processes—including immune response activation, monocyte proliferation, and T-cell activation (38). Notably, this axis promotes an immunosuppressive tumour microenvironment (38). This is a key strategy for CSCs to evade immune surveillance—they often sustain their stemness niche by fostering immune suppression. This axis also shows stronger prognostic value than traditional markers—including B-cell clonality, T-cell clonality, and TMB (38). This suggests that PIWIL4-driven immune modulation may predict clinical outcomes better than tumour-intrinsic features alone. Li et al. also reported that high PIWIL4 protein levels correlate with greater metastasis risk and poorer prognosis in CRC patients (37), reinforcing its functional relevance in CRC progression. Using the GDSC and CTRP databases, Ren et al. identified drugs that may be sensitive to PIWIL4 expression. They found that sensitivity to mTOR inhibitors—INK-128, entosplenitib, and everolimus—correlates with PIWIL4 levels (38), offering potential options for PIWIL4-targeted therapy in CRC. Direct evidence for PIWIL4 regulating CSC stemness in CRC is not as strong as in RB or ovarian cancer. However, promoting an immunosuppressive microenvironment is itself a key way CSCs sustain their niche. Thus, the LINC00857/PIWIL4 axis can be seen as an indirect reflection of PIWIL4’s stemness function—extended to the microenvironment level.

#### 3.1.5 PIWIL4 in metabolic adaptation and microenvironmental remodelling

Tumour cells rely on metabolic reprogramming and inflammatory microenvironment remodelling to cope with nutrient stress, escape immune attack, and fuel malignant progression (61, 62). In prostate cancer, HCC, and cholangiocarcinoma (CCA), PIWIL4 contributes to metabolic adaptation and microenvironment control. It does so by epigenetically silencing antioxidant defence genes, modulating non-coding RNA-mediated metabolic networks, and reshaping the immune microenvironment (**Table 3**). PIWIL4’s effects on metabolism vary across cancers—from directly creating metabolic vulnerabilities to indirectly modulating metabolic networks.

##### 3.1.5.1 The PIWIL4/piR-31470 complex mediates oxidative stress adaptation in prostate cancer

Glutathione S-transferase π1 (GSTP1) is a major antioxidant enzyme. Hypermethylation of its promoter CpG island—leading to GSTP1 inactivation—is one of the earliest and most frequent events in prostate cancer, occurring in over 90% of cases (61, 63). Zhang et al. uncovered a key role for PIWIL4 in this process (61). Mechanistically, piR-31470—highly expressed in prostate cancer cells—binds PIWIL4 to form a complex. This complex uses sequence complementarity to recognize nascent GSTP1 RNA transcripts. It then recruits DNMT1, DNMT3α, and MBD2 to the GSTP1 promoter. These proteins initiate and sustain CpG island methylation, ultimately silencing GSTP1 (61). GSTP1 loss makes cells more prone to oxidative stress and DNA damage (61). *In vitro* experiments confirmed this: overexpressing piR-31470 in RWPE1 cells suppressed GSTP1 and increased their vulnerability to oxidative stress and DNA damage (63). This mechanism has a positive feedback loop. Oxidative stress from GSTP1 loss can trigger DNA damage and genomic instability, which in turn fuels clonal evolution. Given that GSTP1 loss is a hallmark of prostate cancer, the PIWIL4/piR-31470 complex that drives it emerges as an early and critical node in prostate cancer development. Kocic et al. further outlined a broader framework linking immune evasion and metabolic reprogramming. In advanced prostate cancer, PIWIL4 downregulation coincides with accelerated degradation of nucleic acid-sensing TLR ligands (TLR3, TLR7/8, TLR9), allowing immune escape. At the same time, purine/pyrimidine salvage pathways activate, supplying nucleotide metabolism to tumour cells (62). NF-κB activation in turn reshapes the inflammatory microenvironment (62), connecting PIWIL4’s metabolic role to inflammatory remodelling (**Tables 2**, **3**).

**Table 2:** PIWIL4-driven metabolic adaptation and microenvironmental remodelling in prostate cancer.

Analysis dimension	PIWIL4/piR-31470-GSTP1 axis	Downregulation of PIWIL4 + microenvironmental remodelling
PIWIL4 status	High expression of PIWIL4, actively driven	PIWIL4 is downregulated, accompanied by pathway inactivation
Core Effect	GSTP1 apparent silencing → increased susceptibility to oxidative stress	Nucleic acid ligand degradation → evasion of immune surveillance; nucleotide recycling → metabolic support
Functional consequences	Accumulation of DNA damage → genomic instability → progression-driving	Inflammatory remodelling + metabolic adaptation → malignant transformation
Time period	The early stages of prostate cancer	Advanced-stage prostate cancer

→, indicates causal or sequential biological progression.

##### 3.1.5.2 ceRNA networks drive PIWIL4-mediated metabolic reprogramming in hepatocellular carcinoma

In HCC, PIWIL4 regulates metabolism through a fundamentally different mechanism than in prostate cancer. Instead of direct piRNA-guided methylation, it acts indirectly via competitive endogenous RNA (ceRNA) networks (32–36). Huang et al. performed a multi-omics analysis and found that PIWIL1 and PIWIL4 are overexpressed in mouse liver cancer tissues, while PIWIL2 is downregulated (32). Mechanistically, certain lncRNAs—Pvt1, Gas5, and BGIGI10090_38749—act as sponges that bind miR-351-5p and miR-31-5p. This relieves miRNA-mediated suppression of *PIWIL1* and *PIWIL4*, boosting their expression (32). Elevated PIWIL4 also interacts with RNGS (regulatory non-coding RNA-mediated gene silencing) pathway proteins—Dhx9, Drosha, Mov10, and Tdrd1—and participates in RNA binding, gene silencing, and metabolic regulation (32). Functional enrichment analysis painted a clearer picture (32). Downregulated miRNA targets were mainly enriched in lipid metabolism, complement activation, bile secretion, and amino acid biosynthesis. Upregulated targets, in contrast, were enriched in pathways that support tumour growth and invasion—translation, cytoskeletal organization, glycolysis, and ribosome biogenesis (32) (**Table 3**). This bidirectional pattern reflects PIWIL4’s dual role in HCC metabolic reprogramming. On the one side, it dampens host anti-tumour defences by suppressing lipid metabolism and immune pathways. On the other side, it fuels rapid tumour cell growth by boosting glycolysis and protein synthesis (32). Of note, PIWIL4 also has physiological roles in liver regeneration. Rizzo et al. found that after partial hepatectomy, PIWIL4-associated piRNAs show temporal changes that match hepatocyte entry into S phase. This suggests PIWIL4 participates in normal cell cycle control and hepatic metabolic adaptation (36). Its tumour-related metabolic functions may arise from ‘hijacking’ this normal programme (**Figure 3**).

**Figure 3:**
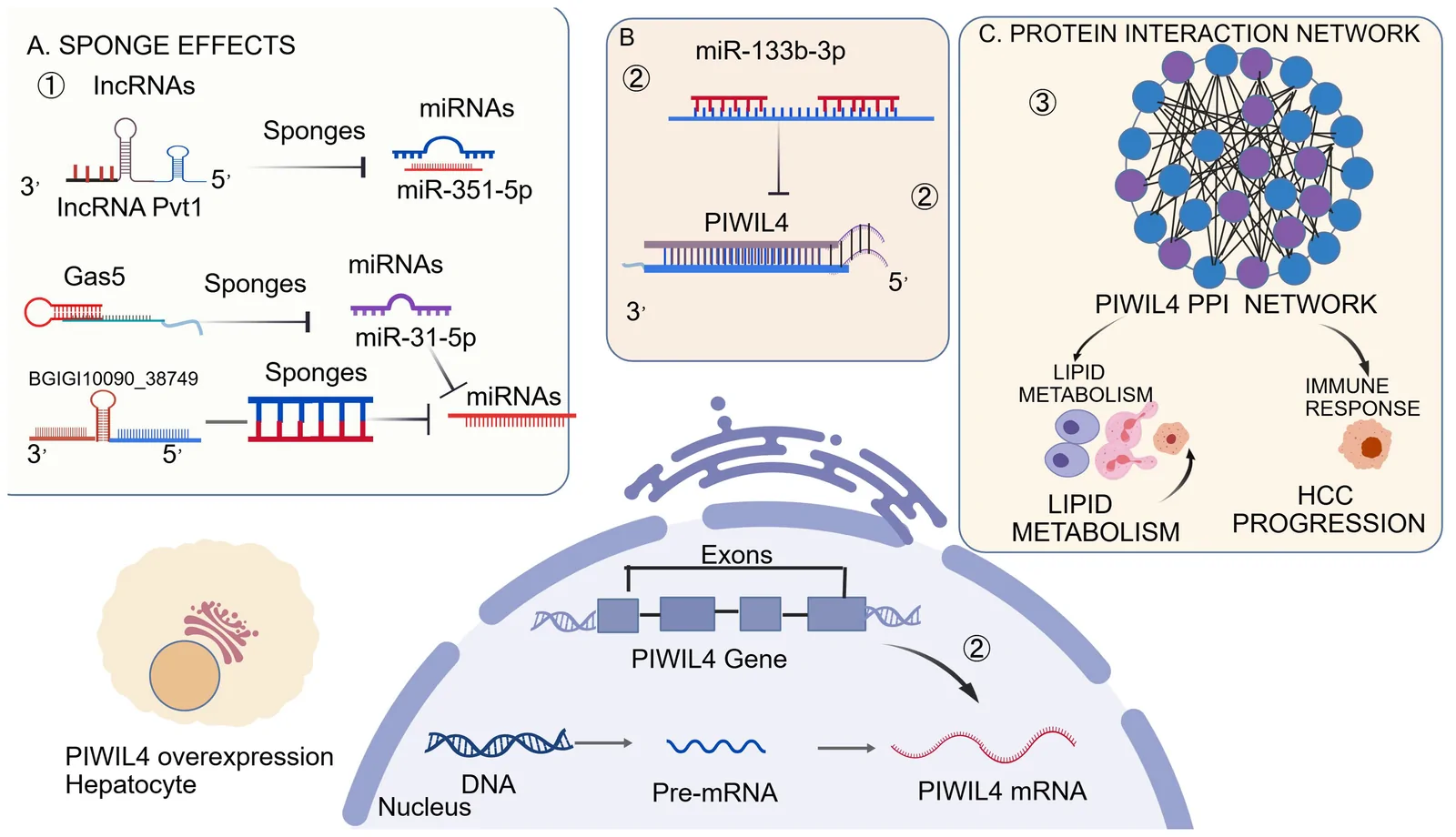
PIWIL4 is controlled by layered post-transcriptional mechanisms and protein interaction networks that drive HCC progression. **(A)** lncRNA-mediated sponge effect (ceRNA mechanism). *Pvt1 and Gas5* act as competitive endogenous RNAs (ceRNAs). They share a miRNA response element and sponge microRNAs, relieving miRNA-mediated silencing of *PIWIL4* mRNA and boosting PIWIL4 protein levels. **(B)** An example of a miRNA-mediated post-transcriptional repression mechanism: miR-133b-3p binds to PIWIL4 mRNA and inhibits its translation; the central section illustrates the transcription and mRNA maturation processes of the PIWIL4 gene. **(C)** PIWIL4 interaction network and downstream metabolic reprogramming. When overexpressed, PIWIL4 recruits various oncogenic effectors through direct or indirect interactions. This activates lipid metabolism pathways (fatty acid synthesis, desaturation, and uptake) which supply the energy and membrane building blocks needed for HCC cell proliferation, survival, and metastasis. Together, lncRNA-mediated de-repression and the PIWIL4-centred interaction network form a pro-tumour axis. This axis links non-coding RNA circuits to metabolic reprogramming. Thus, PIWIL4 emerges as a key node connecting RNA regulation and metabolic remodelling in HCC. Arrows denote activation or positive regulation. T-shaped lines denote inhibition. Solid lines represent validated interactions; dashed lines indicate multi-step or indirect effects.

##### 3.1.5.3 PIWIL4 is part of a metabolism-related prognostic network in Cholangiocarcinoma

PIWIL4 is differentially expressed in cholangiocarcinoma, as reported in multiple studies (64, 66, 67). Most evidence connecting PIWIL4 to metabolic adaptation in this cancer comes from prognostic biomarker studies (65). Using the TCGA (The Cancer Genome Atlas) and GSE107943 (Gene Expression Omnibus) cohorts, Xu et al. developed a prognostic model that includes PIWIL4 and CFHR3 (mRNAs), along with AC007285.1 and AC134682.1 (lncRNAs) (65). This model stratifies patient risk independently of clinical features (HR = 0.20, P = 3.83 × 10^−2^). It has an AUC of 0.781 for predicting 3-year overall survival (OS) (65). Mechanistically, weighted gene co-expression and enrichment analyses showed that this model correlates significantly with metabolism-related processes and lipid metabolism pathways (65). Given that lipid metabolism plays a key role in cholangiocarcinoma development and progression, PIWIL4’s presence in this model hints that it may affect patient prognosis by modulating lipid metabolism genes (**Table 3**).

**Table 3:** summarizes PIWIL4’s roles in metabolic adaptation and microenvironmental remodelling across selected tumours.

Analysis dimension	Prostate cancer	HCC	CAA
PIWIL4 mode of action	Active regulation (piRNA-guided targeted methylation)	Network involvement (ceRNA regulation + Protein-Protein Interaction (PPI) interactions)	Online engagement (component of the prognostic model)
Key metabolic targets	GSTP1 (antioxidant defence)	Lipid metabolism, glycolysis, amino acid metabolism	Lipid metabolism pathways
Mechanism of action	Apparent silencing → Increased susceptibility to oxidative stress	Bidirectional regulation of miRNA target genes → metabolic reprogramming	Influencing metabolic genes via co-expression networks
PIWIL4 status	High expression (early driver)	Overexpression (promoting the progression of HCC)	High expression (protective prognostic factor)
Upstream regulation	piR-31470 guide	lncRNA acts as a sponge to adsorb miRNA	Not specified

→, indicates causal or sequential biological progression.

#### 3.1.6 PIWIL4 maintains genomic stability as an R-loop helicase in AML—a non-classical function

AML ranks among the cancers with the highest PIWIL4 expression (68). In contrast to its piRNA-dependent epigenetic role in other cancers, PIWIL4 in AML takes on a non-classical, piRNA-independent function: it acts as an R-loop helicase to preserve genomic stability. This finding has shifted how we view PIWI protein function (27).

##### 3.1.6.1 PIWIL4 overexpression in AML is driven by leukaemia stem cells

Bamezai et al. found that PIWIL4 is markedly overexpressed in AML patients—at levels higher than in any other cancer type (69). Of note, PIWIL4 is highly expressed in leukaemia stem cells (LSCs) and their progeny. However, it is dispensable for maintaining healthy haematopoietic stem and progenitor cells (HSPCs)—depleting PIWIL4 does not affect their colony formation, differentiation, or *in vivo* engraftment. Knocking down or knocking out PIWIL4 (via shRNA or gRNA) markedly reduced colony formation and liquid culture proliferation in AML cell lines. It also severely impaired LSC transplantability. In limited-dilution assays, PIWIL4 depletion cut LSC frequency by 39-fold. MYC and other oncogenes can bind the PIWIL4 promoter and drive its overexpression—explaining the high PIWIL4 levels seen in AML (28).

##### 3.1.6.2 PIWIL4 acts as an R-loop helicase—a non-classical enzymatic function

A key discovery by Bamezai et al. concerns how PIWIL4 works (28). PAR-CLIP experiments showed that in AML cells, PIWIL4 binds only 6% of known piRNAs. Instead, nearly half (48%) of PIWIL4-bound RNAs map to protein-coding genes, and 17% to enhancer regions. These targets are strongly enriched in cancer-related genes and human myeloid progenitor cell signatures. Mechanistically, PIWIL4 belongs to the RNase H-like superfamily and acts as an R-loop helicase in AML cells. R-loops are RNA-DNA hybrids that form when nascent RNA transcripts hybridize with template DNA strands. They may account for 5–10% of the genome. R-loops serve some physiological roles in transcription and DNA repair. However, their abnormal buildup can stall transcription, cause replication stress, and trigger DNA damage. In AML cells, PIWIL4 specifically binds and clears R-loops that accumulate at genes linked to LSC stemness—including human myeloid progenitor markers and LSC-associated genes. This ensures normal transcriptional elongation at these loci. When PIWIL4 is depleted, R-loops build up at these key loci. This stalls transcription, causes DNA damage and replication stress, and ultimately activates the ATR pathway. The result: loss of LSC stemness and cell apoptosis (28).

##### 3.1.6.3 A therapeutic window: PIWIL4 depletion synergizes with ATR inhibition

Because PIWIL4 is essential for LSCs but not for healthy HSPCs, this creates a therapeutic window for selective targeting in AML (28). The study showed that depleting PIWIL4 markedly increases AML cell sensitivity to ATR inhibitors. This offers a synthetic lethal combination strategy for AML treatment. Interestingly, the link between PIWIL4 expression and AML prognosis cuts both ways. High PIWIL4 indicates active LSCs and correlates with higher white blood cell counts and LDH levels. Yet AML patients with high PIWIL4 also show better outcomes—higher complete remission rates after induction chemotherapy (82.6% vs. 71.7%) and longer overall survival (median 52.8 vs. 17.8 months). This apparent paradox suggests that high PIWIL4 marks an AML subtype that is more chemosensitive. Thus, its value as a therapeutic target may depend on the treatment context. Moreover, treating THP-1 monocytes with differentiation inducers markedly reduced PIWIL4 expression. This suggests PIWIL4 helps maintain the undifferentiated state of myeloid cells—a role that echoes its classical function in preserving germ cell stemness (28). **Table 4** compares the roles of PIWIL4 in AML versus solid tumours.

**Table 4:** PIWIL4 mechanisms in AML versus solid tumours.

Analysis dimension	AML	Solid tumours (gastric cancer, prostate cancer, etc.)
PIWIL4 functional modes	Non-classical (R-loop helicase)	Classic (piRNA-mediated epigenetic regulation)
piRNA-dependent	Virtually no reliance on	Highly dependent
Core molecular events	Eliminating R-loops → Maintaining genomic stability	Apparent silent target genes → regulation of proliferation/apoptosis/EMT
Cell-type specificity	LSCs depend on it, whereas normal HSPCs do not	Both tumour cells and normal cells are affected
Treatment strategies	PIWIL4 inhibitor + ATR inhibitor (synthetic lethality)	PIWIL4 monotherapy

→, indicates causal or sequential biological progression.

### 3.2 PIWIL4 can also act as a tumour suppressor—mechanisms and evidence

In solid tumours, PIWIL4 does more than just promote cancer. Accumulating evidence shows that in certain tissue contexts, PIWIL4 can suppress tumours. It may do so by triggering oxidative stress, activating the DNA damage response and p53 pathway, or simply reflecting a well-differentiated phenotype. This functional reversal provides the strongest evidence for the context-dependent nature of PIWIL4.

#### 3.2.1 The reactive oxygen species/DNA damage/p53 axis mediates PIWIL4’s tumour-suppressive effect in fibrosarcoma

Fibrosarcoma offers a striking contrast. In this cancer, PIWIL4 triggers G_2_/M arrest just as it does in gastric cancer—but here the outcome is apoptosis, not proliferation. Das et al. showed that PIWIL4 suppresses fibrosarcoma through the ROS/DNA damage/p53 axis (49). Mechanistically, overexpressing PIWIL4 triggers a marked buildup of ROS inside cells. This in turn causes genomic DNA damage, seen as increased γH2AX foci. This damage signal activates p53, boosting p53 and its downstream effector p21. The result is G_2_/M arrest and apoptosis (49). This is strikingly different from its effect in gastric cancer, where PIWIL4 also induces G_2_/M arrest but promotes proliferation (29). This ‘same checkpoint, opposite fates’ scenario is central to understanding PIWIL4’s functional duality. In gastric cancer, upstream signalling defects often inactivate p53, so G_2_/M arrest fails to trigger apoptosis (29). In fibrosarcoma, however, p53 is intact, allowing ROS/DNA damage signals to effectively reach the apoptotic machinery. Das et al. confirmed this: in fibrosarcoma cells with functional p53, overexpressing PIWIL4 suffices to trigger apoptosis. This tumour-suppressive effect, however, hinges on p53 pathway integrity (41). This finding clarifies how PIWIL4 suppresses tumours and highlights its potential as a predictive biomarker. p53 status may shape the outcome of PIWIL4-targeted therapy. In p53-wild-type tumours, activating PIWIL4 could be beneficial; in p53-mutant ones, inhibiting it would be necessary.

#### 3.2.2 Clinical evidence links PIWIL4 to favourable prognosis in some cancers

Unlike the well-defined tumour-suppressive mechanisms in fibrosarcoma, the evidence for PIWIL4’s tumour-suppressive role in renal cell carcinoma (RCC), soft tissue sarcoma (STS), and some lung cancers comes mainly from clinical prognostic correlations and expression studies. The underlying mechanisms, however, remain unclear. While these findings are less robust than the mechanistic data from fibrosarcoma, they still offer important clinical evidence for PIWIL4’s dual functionality.

##### 3.2.2.1 In RCC, high PIWIL4 expression correlates with better prognosis

Al-Janabi et al. measured PIWI family gene transcripts in RCC and found PIWIL4 markedly overexpressed in tumour versus normal tissue (42, 43). Iliev et al. confirmed this in an independent cohort and further showed that RCC patients with high PIWIL4 had longer overall survival (OS) (43). Multivariate analysis identified PIWIL4 as an independent protective prognostic factor in RCC. Several explanations are possible. (i) Tumours with high PIWIL4 may behave more indolently. (ii) PIWIL4 might slow RCC progression through unknown mechanisms—such as modulating immune infiltration or differentiation. (iii) High PIWIL4 may simply mark a well-differentiated phenotype, not directly suppress tumours. Whatever the explanation, the link between high PIWIL4 and good prognosis in RCC contrasts sharply with the high PIWIL4–poor prognosis link in gastric cancer. This clearly illustrates how PIWIL4’s function reverses across tissue contexts.

##### 3.2.2.2 In STS, low PIWIL4 expression predicts poor prognosis

Greither et al. assessed the prognostic value of PIWI-like genes in STS patients and found that low PIWIL4 mRNA levels correlate with poor outcomes (1). They validated this in a cohort of 125 STS patients. Of note, they also reported that gender influences PIWIL4’s prognostic value. PIWIL4 mRNA levels predict survival in male STS patients but not in females (1). The reason for this gender difference is unclear, but future clinical use of PIWIL4 biomarkers should consider sex stratification (1).

##### 3.2.2.3 In some lung cancer subgroups, low PIWIL4 expression predicts recurrence

Navarro et al. found that in non-small cell lung cancer, PIWIL4 is markedly downregulated in tumour versus normal tissue. Patients with low PIWIL4 had shorter time to recurrence (TTR) and OS (54). At first glance, this seems to conflict with the PLIC11/YY1/PIWIL4 oncogenic axis reported by Zhu et al. (53). But it may actually reflect a functional switch across lung cancer subtypes or stages. In early-stage or certain molecular subtypes, PIWIL4 may maintain normal epithelial differentiation and genomic stability. Losing it could create conditions for malignant transformation (tumour-suppressor mode). In advanced stages or other subtypes, however, PIWIL4 gets hijacked by aberrantly activated transcriptional axes (like PLIC11/YY1) and turns into an oncogenic driver. This ‘stage-specific switch’ hypothesis needs testing in longitudinal cohort studies and single-cell analyses. WeiLi et al. also included PIWIL4 in several risk models linked to prognosis (55), reinforcing its complex role in lung cancer outcomes.

### 3.3 The molecular basis of PIWIL4’s functional duality and its switching mechanism

As discussed above, PIWIL4 can produce different outcomes even at the same cell cycle checkpoint (e.g., G_2_/M). This raises a key question: what determines which path PIWIL4 takes? Based on current evidence, we propose three levels of regulation. First, PIWIL4 function depends on which piRNA it binds. Unlike *PIWIL2*, which mainly binds piRNAs from repetitive sequences, *PIWIL4* associates with a broader range of piRNAs. For example, *piR-31115* guides PIWIL4 to HSP90AA1 in breast cancer (40); *piR-31470* directs it to the GSTP1 promoter in prostate cancer (61); and CHAPIR mediates PIWIL4–METTL3 interaction in cardiac hypertrophy (70). Different piRNAs thus shape where PIWIL4 binds on chromatin and which proteins it interacts with—ultimately changing its functional output. However, systematic comparisons of PIWIL4-bound piRNA profiles across tumours are still lacking. Second, PIWIL4 activity is finely tuned by its interacting partners. In gastric cancer, PIWIL4 teams up with PIWIL2 to drive tumour progression (29, 44). In AML, it works with SUPT5H to sustain leukaemia stem cell stemness (67). In prostate cancer, it recruits DNMT1 and DNMT3α to the GSTP1 promoter (61). And during HIV-1 latency, it recruits HP1α/β/γ, SETDB1, and HDAC4 to form a repressive complex (71). These varied partnerships may reflect cell-type-specific expression patterns or differences in PIWIL4’s post-translational modifications. Third, the cellular context itself matters. PIWIL4’s final output depends on the cell’s internal state—p53 pathway integrity, redox balance, and DNA repair capacity. In fibrosarcoma, PIWIL4 overexpression triggers ROS buildup, activates an intact p53 pathway, and induces apoptosis (41). In most cancers, however, p53 is often disabled [e.g. p14ARF suppression in cervical cancer (31)], so PIWIL4’s pro-proliferative effects take over. This hints that PIWIL4 might act as a ‘stress sensor’—triggering apoptosis when p53 is functional, but promoting proliferation when p53 is disabled. This idea deserves further testing. In short, PIWIL4’s functional duality is not a contradiction. It reflects the protein’s context-dependent nature—its output arises from the interplay of piRNA guidance, protein interaction networks, and cellular state.

PIWIL4 functions extend beyond oncology. It also participates in key physiological and pathological processes outside cancer—including stem cell maintenance, reproductive development, neurodegeneration, and inflammatory responses. Here we review recent findings on PIWIL4 in non-cancerous conditions. The following sections detail PIWIL4’s expression patterns and biological functions in these conditions. **Table 5** summarizes this information.

**Table 5:** Molecular mechanisms underlying the dual functions of PIWIL4 (tumour promotion vs. tumour-suppressive).

Functional focus	Representative types of cancer	Core mechanism pathway	Upstream activation signal	Keydown stream effect molecules	Cell fate outcomes	Level of evidence	Ref.
Tumer-promoting	Gastric Cancer	G2/M checkpoint escape	High expression of PIWIL4	Cyclin/CDK regulatory factors	Proliferation↑	Clinical + Functional	(29, 30)
Tumer-promoting	Cervical Cancer	Inhibition of the p14ARF/p53 pathway	High expression of PIWIL4	p14ARF↓,p53↓	Apoptosis ↓, Invasion ↑	Clinical + Functional	(31, 46–48)
Tumer-promoting	Breast Cancer(ER+)	The oestrogen/ERα-EMT axis	Oestrogen/ERα signalling	E-cadherin↓,Vimentin↑	Proliferation↑, migration↑, invasion↑	Clinical + Functional	(72)
Tumer-promoting	Breast Cancer(TNBC)	piRNA-HSP90AA1 axis	piR-31115	HSP90AA1↑, TGF-β/MAPK/ERK pathway	Migration↑	Functional	(23, 24, 52)
Tumer-promoting	Pancreatic Cancer	Activation of the EMT transcription programme	PIWIL3/PIWIL4 co-regulation	E-cadherin↓,Vimentin↑	Metastasis↑, invasion↑, drug resistance↑	Clinical + Functional	(39, 40)
Tumer-promoting	HCC	ceRNA networks – metabolic reprogramming	lncRNA acts as a sponge to bind miR-351-5p/miR-31-5p	Lipid metabolism ↓, glycolysis ↑	Proliferation↑, immune evasion	Functional	(32–36, 64)
Tumer-promoting	AML	R-loop helicase (non-classical function)	Oncogene-driven(MLL-AF9,AML1-ETO9A)	ATR pathway, LSC inactivating gene	LSC self-renewal↑, genomic stability↑	Functional	(27, 28)
Tumer-promoting	Prostate Cancer (Early Stage)	Apparent silencing of GSTP1	piR-31470 guide	DNMT1/DNMT3α,MBD2,GSTP1↓	Oxidative stress ↑, DNA damage ↑, genomic instability	Functional	(61)
Tumer-promoting	Prostate Cancer (Progressive stage)	Immune evasion – metabolic reprogramming	PIWIL4 is downregulated; the miRNA pathway is silenced	DNase II↑, RNase 2↑, NF-κB↑, salvage synthesis pathway↑	Immune surveillance evasion, metabolic adaptation	Clinical + Functional	(62)
Tumer-promoting	Colorectal Cancer	The LINC00857/PIWIL4 immunosuppressive axis	LINC00857 has been raised	Immunosuppressive microenvironment, ↓ T-cell activation	Immune evasion, metastasis ↑	Features	(37, 38, 88)
Tumer-promoting	RB	PIWIL4/piRNA-OTX2 Dry Axis	piRNA-guided	OTX2, chromatin accessibility ↑, differentiation genes ↓	CSC self-renewal↑, differentiation arrest	Features	(25, 26)
Tumer-promoting	Ovarian Cancer	OCSC dry remission – chemotherapy resistance	The PIWIL4/piRNA pathway	Tumour suppressor genes ↓, Golgi-related pathways	Self-updating↑, platinum resistance	Clinical + Functional	(58, 59)
Tumer-promoting	Lung Cancer	PLIC11-YY1-PIWIL4 transcriptional axis	PLIC11 → YY1 activation	Transcriptome reprogramming	Proliferation↑, invasion↑, metastasis↑	Features	(53)
Tumer-promoting	Cholangiocarcinoma	4-RNA Prognostic Model – Lipid Metabolism	Not fully explained	Lipid metabolism pathways	Poor prognosis (low expression)	Clinical	(65–67)
Tumer-promoting	Headand Neck SCC	The HPV-piRNA-PIWIL4 axis	HPV infection → piRNA abnormalities	RTL family genes	Tumour initiation↑, progression↑	Features	(72)
Tumer-promoting	Leydig Cell Tumour	Resistance to the mitochondrial apoptotic pathway	Anti-apoptotic function of PIWIL4	Mitochondrial membrane potential ↑, caspase-9/caspase-3 ↓	Apoptose ↓	Features	(51)
Tumer-suppressive	Fibrosarcoma	ROS/DNA damage/p53 axis	PIWIL4 overexpression	ROS↑,γH2AX↑,p53↑,p21↑,caspase-3↑	G2/M arrest, increased apoptosis	Features	(41)
Tumer-suppressive	Renal Cell Carcinoma	Prognostic protective factors (mechanism to be elucidated)	High expression of PIWIL4	Not fully explained	Overall survival ↑	Clinical	(42, 43)
Tumer-suppressive	Soft Tissue Sarcoma	Prognostic protective factors (mechanism to be elucidated)	High expression of PIWIL4 (protective)	Not fully explained	Overall survival ↑ (female-specific)	Clinical	(1)
Tumer-suppressive	Lung Cancer (certain subgroups)	Prognostic protective factors (mechanism to be elucidated)	High expression of PIWIL4 (protective)	Not fully explained	Recurrence-free survival ↑, overall survival ↑	Clinical	(54, 55)
Tumer-suppressive	Bladder Cancer	Non-invasive diagnostic markers (mechanism to be elucidated)	PIWIL4 is downregulated in tissues and urine	Not fully explained	Increased risk of tumour recurrence (low expression)	Clinical	(86)

↑, increased or enhanced molecular level or biological phenotype; ↓, decreased or repressed molecular level or biological phenotype; →, denotes causal or sequential biological events.

## 4 PIWIL4 functional networks in non-cancerous diseases

Just as PIWIL4 shows diverse functions across cancers, it also plays varied regulatory roles in non-cancerous diseases. Across diverse contexts—from transposon silencing in germ cells to neural differentiation and metabolic homeostasis—PIWIL4 follows a conserved molecular logic. It regulates specific gene programmes by recognizing targets via piRNAs and recruiting epigenetic effectors. Yet its biological outcomes differ widely, depending on which genes are targeted and the cell’s internal state. The following sections are organized around functional theme. Based on this understanding, the following discussion is organised by functional theme and presented visually in **Table 6**.

**Table 6:** The functional roles and core mechanisms of PIWIL4 in non-neoplastic diseases.

Common conditions	The functional role of PIWIL4	Core Mechanism	Ref.
Cryptorchidism	Guardians of the Genome	PIWIL4, in collaboration with GTSF1 and other factors, mediates the epigenetic silencing of LINE-1 retrotransposons via the PIWI-piRNA pathway; dysregulation of this pathway leads to LINE-1 desilencing, inducing germ cell apoptosis and defects in spermatogenesis	(10, 11)
NOA	Guardians of the Genome	A p.R269W mutation in the PAZ domain of PIWIL4 impairs piRNA binding → runaway activity of LINE-1 and other transposons → abnormal DNA methylation → disruption of the spermatogenesis transcriptome; the 3’ UTR rs508485 polymorphism influences disease susceptibility by regulating PIWIL4 expression via miRNAs	(11–15)
CVDs	Protective factors	PIWIL4 regulates colour perception and maintains retinal pigment epithelium (RPE) homeostasis via the piRNA pathway	(21)
AMD	Protective factors (mechanism-related)	Oxidative stress localises PIWIL4 to cytoplasmic stress granules → lifting of Alu RNA silencing → abnormal accumulation of Alu RNA → disruption of RPE epithelial characteristics	(22)
wAMD	Pathogenic factors	Upregulation of PIWIL4 → promotes VEGF secretion and enhances VEGFR2 phosphorylation → activates angiogenesis signalling; piRNAs target angiogenesis-related genes	(73)
Neurodevelopment	Developmental regulatory factors	PIWIL4 maintains the expression of stem cell genes such as OCT4 and SOX2, thereby sustaining an undifferentiated state; during differentiation, PIWIL4 is downregulated → leading to the release of differentiation genes such as β-III tubulin and MAP2 → promoting neuronal maturation	(74)
sALS	Pathogenic factors	Abnormal PIWIL4 expression → Dysregulation of the piRNA/PIWI pathway → Transposon activation + DNA methylation imbalance → Accelerated neurodegeneration	(75)
Diabetes	Protective factors	The PIWIL4/piRNA complex maintains β-cell insulin secretion and anti-apoptotic capacity through epigenetic modifications, such as DNA methylation, and post-transcriptional regulation; overexpression of specific piRNAs leads to defects in insulin secretion	(20)
DR	Pathogenic factors	High glucose/oxidative stress/VEGF-induced upregulation of PIWIL4 → regulation of VEGF, growth factors and EMT markers → retinal neovascularisation and disruption of the blood-retinal barrier	(76)
cardiac hypertrophy	Pathogenic factors	The CHAPIR-PIWIL4 complex binds to METTL3, inhibiting its methyltransferase activity → ↓ m^6^A modification of Parp10 mRNA → ↓ Parp10 → inhibition of the anti-hypertrophic pathway	(70)
HIV-1 latency	People with latent viral infection	PIWIL4 binds to the HIV-1 5’ LTR → recruits HP1α/β/γ, SETDB1 and HDAC4 → H3K9me3+ histone deacetylation → heterochromatin formation → viral transcriptional silencing	(71)
teratozoospermia	To be clarified	PIWIL4 is involved in the pathogenesis; the specific molecular targets and signalling pathways remain to be elucidated	(77)
Depression	To be clarified	PIWIL4 is involved in the pathogenesis; the specific molecular targets and signalling pathways remain to be elucidated	(78)
Rheumatoid arthritis	To be clarified	PIWIL4 is involved in the pathogenesis; the specific molecular targets and signalling pathways remain to be elucidated	(79)
gestational hypertensive disorders	To be clarified	PIWIL4, the major isoform in the human placenta, is up-regulated in gestational hypertension	(80)

→, causal or sequential biological events.

### 4.1 Protecting the germline genome: transposon silencing and spermatogenesis

In male germ cells, PIWIL4 carries out its classic genome-guardian role: it silences transposons through the PIWI/piRNA pathway to preserve germline genome integrity. Hadziselimovic et al. found that in cryptorchidism, PIWIL4 works with GTSF1 and other molecules to epigenetically silence LINE-1 retrotransposons. When this pathway fails, LINE-1 becomes derepressed, triggering germ cell apoptosis and spermatogenic defects. This provides a molecular explanation for infertility linked to cryptorchidism (9, 10). In non-obstructive azoospermia (NOA), Wang et al. recently identified a p.R269W mutation in PIWIL4’s PAZ domain. This mutation severely weakens piRNA binding, causing LINE-1 and other transposons to become dysregulated, along with abnormal DNA methylation. The result is a disrupted spermatogenic transcriptome (11). Gonzalez-Munoz et al. further showed that the rs508485 polymorphism in the *PIWIL4* 3’ UTR can modulate PIWIL4 expression by altering miR-215 and miR-136 binding affinity. This in turn affects NOA susceptibility (12). This finding—the first of its kind—directly connects post-transcriptional regulation of PIWIL4 to genetic risk for male infertility (13–15).

### 4.2 PIWIL4 in the visual system: oxidative stress, angiogenesis, and homeostasis

In the visual system, PIWIL4 drives disease through two distinct routes. One involves oxidative stress-triggered cytoplasmic mislocalisation. The other involves modulating signalling pathways that control pathological angiogenesis.

1. Retinal Pigment Epithelium (RPE) Homeostasis and Oxidative Stress. Hwang et al. found that oxidative stress can trap *PIWIL4* in cytoplasmic stress granules, pulling it away from the nucleus. This relieves PIWIL4’s silencing of Alu RNA, causing Alu RNA to build up abnormally and leading to loss of RPE epithelial traits. This pathway—PIWIL4 sequestration leading to Alu RNA accumulation—plays a key role in oxidative stress-related retinal diseases. It offers a fresh perspective on age-related macular degeneration (AMD): the key driver is functional inactivation of PIWIL4, not changes in its expression level (22).
2. Choroidal neovascularisation (CNV). Ren et al. used a laser-induced CNV mouse model and found that PIWIL4 is upregulated in CNV lesions. It activates downstream angiogenic signals by boosting VEGF secretion and enhancing VEGFR2 phosphorylation. Differentially expressed piRNAs in the CNV model target angiogenesis-related genes, hinting that the PIWIL4/piRNA axis regulates pathological neovascularisation. Mechanistically, PIWIL4’s control of angiogenesis parallels its roles in prostate cancer (modulating oxidative stress via piRNA-guided silencing) and HCC (participating in immune and metabolic regulation through ceRNA networks) (see Section 2.1.5). This points to PIWIL4 as a potential regulatory hub for angiogenesis across diverse pathological settings, acting through distinct molecular mechanisms (73).

### 4.3 The nervous system: from developmental programming to degenerative changes

PIWIL4 plays a dual role in the nervous system, spanning development and neurodegeneration. Its function shifts with the cell’s differentiation state and pathological stimuli. In neurodevelopment, Subhramanyam et al. showed that PIWIL4 is key to keeping embryonic cancer cells (NT2) undifferentiated. Specifically, PIWIL4 regulates *OCT4* (Octamer-binding transcription factor 4) and *SOX2* (SRY-box transcription factor 2) expression via the piRNA pathway, locking cells in an undifferentiated state. When neural differentiation begins, PIWIL4 drops, releasing differentiation-related genes (including β-III tubulin and MAP2) and driving cells toward a mature neuronal phenotype. This finding establishes—for the first time in a human neurodevelopmental model—that the PIWI/piRNA pathway plays a role in cell fate determination. Its mechanism parallels how PIWIL4 maintains stem cell pluripotency in retinoblastoma (Section 2.1.4). This suggests that PIWIL4 may use the same molecular circuit—regulating core transcription factors—to produce very different outcomes depending on the cellular context (74).

In neurodegenerative diseases, Abdelhamid et al. found that patients with sporadic amyotrophic lateral sclerosis (sALS) show abnormal *PIWIL1* and *PIWIL4* expression, along with piRNA/PIWI pathway dysregulation. This leads to transposon activation and DNA methylation imbalance, which in turn accelerates neurodegeneration. This offers a fresh perspective on how non-coding RNA-mediated regulation contributes to sALS pathogenesis. It also raises the possibility that aberrant PIWIL4 activation in mature neurons may engage a transposon silencing programme akin to that in germ cells—yet in neurons, this response triggers genomic stress and degeneration (75).

### 4.4 Metabolic homeostasis: β-cell function, insulin resistance, and diabetic complications

In the metabolic system, PIWIL4 contributes to pancreatic β-cell function and diabetic complications through the piRNA/PIWI pathway. Henaoui et al. first reported piRNA expression profile changes in a rat model of type 2 diabetes. Overexpression of certain piRNAs impaired glucose-stimulated insulin secretion. Mechanistically, PIWIL4 forms complexes with piRNAs and preserves both insulin secretion and anti-apoptotic capacity in β-cells. It does so through epigenetic changes (e.g., DNA methylation) and post-transcriptional regulation. This finding broadens the known functions of the PIWI/piRNA pathway beyond germ cells and tumours into endocrine metabolism. It suggests that the pathway may help regulate stress responses and functional homeostasis in various secretory cell types (20).

In diabetic retinopathy (DR), Sivagurunathan et al. found that a pathological microenvironment—marked by chronic hyperglycaemia, oxidative stress, and high vitreal VEGF can upregulate PIWIL4. PIWIL4 then promotes retinal neovascularisation and disrupts the blood-retinal barrier by modulating VEGF, growth factors, and EMT markers. The upstream triggers differ between DR (hyperglycaemia, oxidative stress) and Wet age-related macular degeneration (wAMD) (laser damage, hypoxia). Yet the downstream outcomes(enhanced VEGF signalling and pathological angiogenesis) are strikingly similar. This suggests that PIWIL4 may serve as a common node across multiple angiogenesis-related diseases (76).

### 4.5 The cardiovascular system: a new dimension of epigenetic regulation through m^6^A modification

In cardiac hypertrophy, PIWIL4 participates in a non-classical epigenetic mechanism—it directly regulates m^6^A RNA modification through piRNAs. Gao et al. identified a hypertrophy-related piRNA (CHAPIR) that binds METTL3 after forming a complex with PIWIL4. This binding specifically inhibits METTL3’s methyltransferase activity—without altering its expression or stability. This lowers m^6^A modification on Parp10 mRNA, which suppresses anti-hypertrophic pathways and ultimately drives cardiac hypertrophy. This reveals a new piRNA paradigm: the piRNA/PIWIL4 complex not only acts as an epigenetic regulator at the DNA level but also controls m^6^A modification by binding directly to RNA-modifying enzymes—thus affecting mRNA stability and translation (70). Together with PIWIL4’s R-loop helicase function in AML (see Section 2.1.6), this mechanism exemplifies PIWIL4’s ‘functional plasticity’—the same core protein is ‘domesticated’ to carry out entirely different tasks in different cellular settings.

### 4.6 Infection and immunity: PIWIL4 as an epigenetic maintainer of HIV-1 latency

In HIV-1 infection, PIWIL4 plays a unique role in regulating the virus. He et al. showed that PIWIL4 binds the HIV-1 provirus at the 5’ long terminal repeat (5’LTR). It then recruits HP1α/β/γ, SETDB1, and HDAC4, which trigger H3K9me3 and histone deacetylation. This promotes heterochromatin formation, silencing LTR-driven transcription and maintaining HIV-1 latency. Functional tests confirmed that Jurkat T cells lacking PIWIL4 are more susceptible to reactivation by latency-reversing agents (LRAs)—including vorinostat, JQ1 (a small-molecule chemical inhibitor), and prostaglandins. This confirms that targeting PIWIL4 or its downstream effectors offers a novel ‘block and clear’ strategy. Of note, PIWIL4’s epigenetic silencing here is molecularly similar to its classical role in silencing germline transposons—both involve H3K9me3 and heterochromatin formation. Yet their biological purposes are opposite: in germ cells, PIWIL4 shields the genome from ‘selfish DNA; in HIV-1 infection, it shields the virus from immune clearance (71).

### 4.7 Other diseases

Abnormal PIWIL4 expression and dysfunction have also been linked to teratozoospermia (77), depression (78), and rheumatoid arthritis (79). As the main isoform in the human placenta, PIWIL4 is upregulated in gestational hypertensive disorders and appears to play a regulatory role there (80). The specific targets and pathways of PIWIL4 in these conditions still need clarification. Nevertheless, its involvement across multiple systems and diseases reinforces its status as a multifunctional regulator—whose output depends on cell type, developmental stage, and pathological stimuli.

## 5 PIWIL4 expression in other animals

PIWIL4 is central to many human diseases, acting through various signalling pathways and pathogenic mechanisms. It participates in diverse biological processes—including cancer epigenetics (81), RNA stability (7), transposon silencing (82–84), and translation repression (85). PIWIL4 also appears to have important functions in other animal species, though the underlying mechanisms remain to be explored. Furthermore, it exhibits racial variation; in terms of gene origin, *PIWIL1* and *PIWIL2* are ancestral genes in vertebrates, and all members have been retained. *PIWIL4* is a duplication product of *PIWIL1* in teleosts, but has been independently lost in birds and certain fish species (11). In Drosophila, mice, rats, rhesus macaques, pigs, Tibetan antelopes and golden ground squirrels, *PIWIL4* plays a role in germline maintenance; its involvement in piRNA-mediated transposon silencing is crucial for spermatogenesis (1, 2, 4, 5, 8, 11). However, a recent study (86) has revealed that PIWIL4 enhances endonuclease activity; it assists the protein GTSF1 in amplifying the weak intrinsic RNA-cleaving activity of PIWI proteins such as PIWIL4, a regulatory mechanism that is evolutionarily conserved. Although PIWIL4 is primarily restricted to the gonads, somatic expression has been detected in mice and rats (lung, erythroid progenitor cells, pancreatic β-cells) (8, 85, 87), suggesting that its functional diversity is evolving. *PIWIL4* is highly conserved in terms of its core functional domains and reproductive functions, but there are significant species differences in its tissue distribution, developmental dynamics, requirements for female fertility, and somatic functions. Rhesus macaques are the closest to humans in terms of sequence and physiological relevance, whilst mice remain the model of choice for mechanistic studies. When selecting a model, the level of study (evolutionary, mechanistic, physiological or pathological) must be clearly defined, and due attention must be paid to the species-specific characteristics of female reproductive and somatic functions.

## 6 Clinical translation potential, challenges, and strategies for PIWIL4

### 6.1 Gaps in the clinical evidence for PIWIL4 as a biomarker

Currently, most evidence for PIWIL4 as a biomarker comes from retrospective IHC analyses of tissue samples—gastric cancer (N = 182) (29) and pancreatic cancer (N = 226) (39), for example. Prospective cohort validation is still missing. More critically, PIWIL4 has yet to transition from a tissue-based marker to a liquid biopsy tool. It shows potential for urine detection in bladder cancer (88), but its sensitivity (not yet reported) and specificity need validation in large cohorts. Three key challenges stand out. (i) Background expression in normal tissues: PIWIL4 is highly expressed in the testes (89), so circulating PIWIL4 or anti-PIWIL4 antibodies could pick up signals from the reproductive system. (ii) Tissue heterogeneity: PIWIL4 expression can vary spatially within a tumour, so a single biopsy may not capture the full picture. (iii) Cut-off standardization: IHC scoring criteria differ across studies, and no universally accepted positive threshold exists.

### 6.2 Drug development strategies and obstacles for PIWIL4-targeted therapies

The main challenge in targeting PIWIL4 therapeutically is the druggability of its functional domains. First, consider the protein–protein interaction interfaces. Both the PAZ and Piwi domains mediate protein–protein and protein–RNA interactions (2–4). These interfaces tend to be flat and hydrophobic, making them traditionally “undruggable”. PROTAC (Proteolysis-Targeting Chimera) technology offers a new way forward. By designing bifunctional molecules that recruit E3 ubiquitin ligases, one can induce targeted PIWIL4 degradation—theoretically bypassing the limits of traditional ‘site-occupying’ inhibitors. Second, small-molecule inhibitor screening. Ren et al. mined the GDSC and CTRP databases for PIWIL4-sensitive drugs and found that sensitivity correlates with mTOR inhibitors—INK-128, entosplenitib, and everolimus (90). These effects, however, are indirect. No small molecule directly targeting PIWIL4 has been reported so far. Structural studies—such as solving the crystal structure of full-length PIWIL4 or its domains—will be key for rational drug design. Third, combination therapy strategies. The most promising approach comes from AML studies: depleting PIWIL4 sensitizes leukaemic cells to ATR inhibitors (28), suggesting that combining PIWIL4 and ATR inhibitors could have synergistic anti-leukaemic effects. Likewise, silencing PIWIL3/PIWIL4 in pancreatic cancer increases chemosensitivity (39), hinting that combining this with gemcitabine deserves further study. Still, these are preclinical observations that need validation *in vivo* and in early-phase trials. Fourth, safety. PIWIL4 is physiologically expressed at high levels in the testis (89) and is essential for spermatogenesis (91, 92). Systemic inhibition could therefore cause reproductive toxicity. Balancing anti-tumour efficacy with reproductive safety is an unavoidable challenge in developing PIWIL4-targeted drugs. Possible solutions include local delivery, antibody-drug conjugates that target tumour-specific antigens, or inhibitors that disrupt tumour-specific PIWIL4 interaction networks—rather than PIWIL4 itself.

### 6.3 A roadmap from bench to bedside

We propose a three-stage roadmap for translating PIWIL4 into clinical practice. (i) Short term: validate its prognostic value in multiple independent prospective cohorts and establish standardized testing. (ii) Medium term: solve the structures of PIWIL4 in complex with key partners—such as SUPT5H (67) and METTL3 (70)—to guide rational drug design. (iii) Long term: develop PIWIL4-PPI small-molecule inhibitors or PROTAC degraders based on structural data, test their efficacy alone and in combination in preclinical models, and monitor reproductive toxicity closely. To better illustrate PIWIL4’s diagnostic and therapeutic potential, we summarize current strategies, mechanisms, and supporting evidence, and highlight its promise for improving patient outcomes (**Figure 4**; **Table 7**).

**Figure 4:**
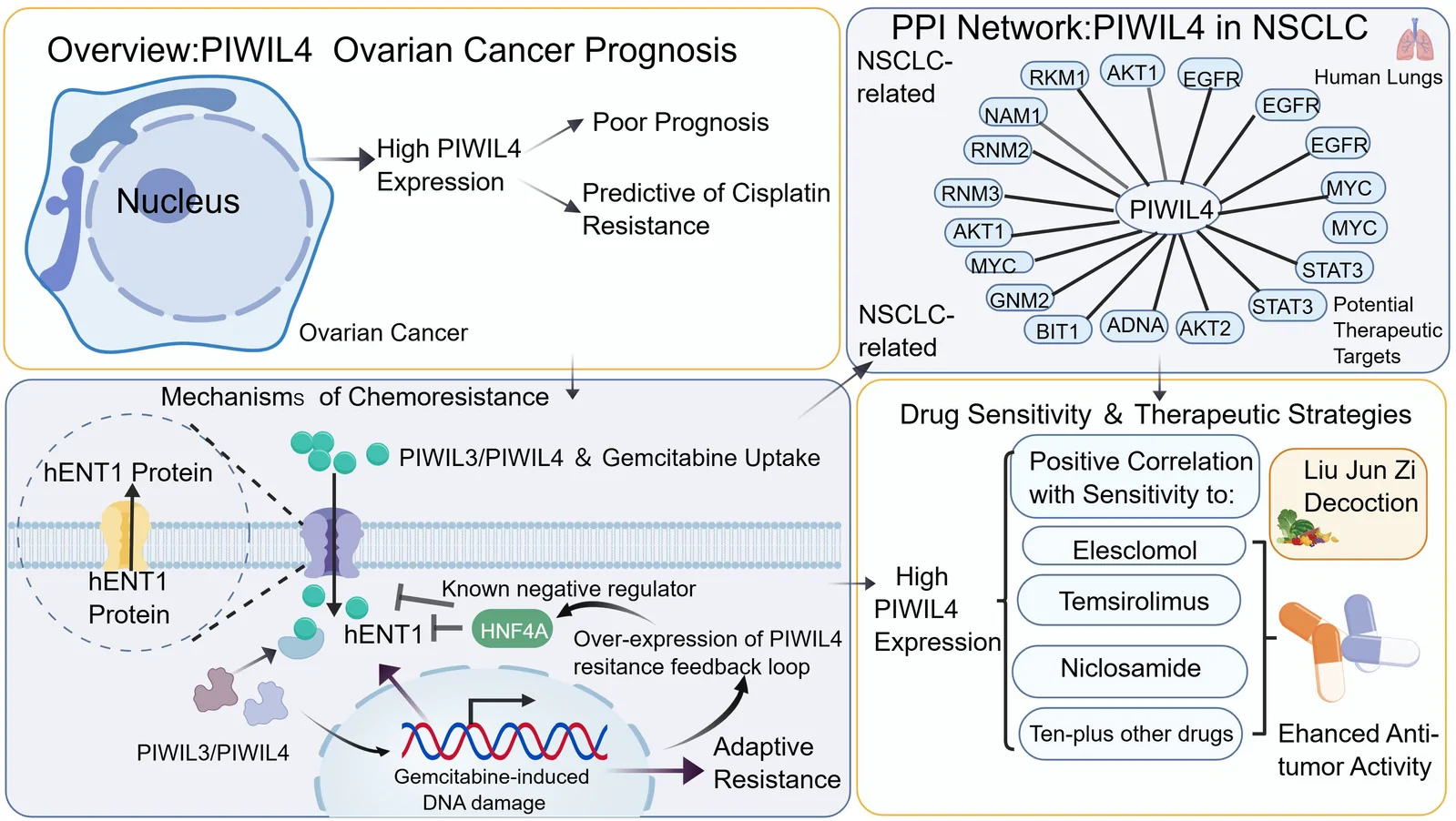
Clinical translation potential of PIWIL4 as a predictive biomarker and therapeutic target in chemotherapy-resistant tumours. In ovarian cancer (left), high PIWIL4 expression correlates with poor platinum-based chemotherapy response. Non-responders had much higher PIWIL4 levels than responders within the 6-month recurrence-free survival period. In pancreatic cancer (right), PIWIL3/PIWIL4 expression links to gemcitabine sensitivity through HNF4A. HNF4A is a negative regulator of hENT1, the transporter that takes up gemcitabine. PIWIL4 mRNA correlates moderately and positively with HNF4A. This correlation may partly explain how PIWIL3/PIWIL4 regulates chemoresistance in pancreatic cancer cells. General graphical conventions: Solid arrows = activation/promotion; bar-ended lines = inhibition; dotted arrows = indirect regulation; straight connecting lines = physical protein-protein interaction.

**Table 7:** Clinical translational applications of PIWIL4: diagnostic and therapeutic potential.

Disease	Core mechanism of action	Clinical sample validation	Clinical trials	Ref.
Gastric Cancer	High expression of PIWIL4 can identify high-risk patients; silencing PIWIL4 inhibits proliferation and induces G2/M phase arrest	Yes(N = 182)	—	(29, 30)
Cervical Cancer	PIWIL4 promotes growth, inhibits apoptosis and enhances invasiveness via the p14ARF/p53 pathway	Yes	HeLa,C33A	(31)
Ovarian Cancer	High expression of PIWIL4 is associated with resistance to platinum-based chemotherapy and may serve as a predictive biomarker for RFS	Yes(N = 20)	A2780,SKOV3	(58, 59)
Pancreatic Cancer	PIWIL4 is positively correlated with HNF4A expression and may mediate gemcitabine resistance via the HNF4A/hENT1 axis	Yes(N = 83–226)	PDAC-derivedcelllines	(39, 40)
Lung Cancer	PIWIL4 is a downstream effector molecule of the PLIC11/YY1 axis; the PPI network involved in the anti-NSCLC activity of LiuJunZi Decoction	Yes	—	(53)
Colorectal Cancer	PIWIL4 is a downstream target gene of LINC00857; PIWIL4-sensitive drugs (INK-128, entosplenitib, everolimus) predicted based on GDSC/CTRP	—	HCT116	(38, 88)
Leydig Cell Tumour	Epimedium glycoside downregulates PIWIL4 expression → activation of the mitochondrial pathway → induction of apoptosis	—	MLTC-1	(51)

## 7 Outstanding issues and future directions

### 7.1 Structural biology: from sequence to three-dimensional structure

Although structures are available for PAZ (3) and Piwi (4) domain homologues, we still lack a complete structure of full-length PIWIL4, as well as high-resolution views of its complexes with piRNAs and partner proteins. Future efforts should focus on: (i) determining the PIWIL4–piRNA–target DNA/RNA ternary complex structure by cryo-EM and X-ray crystallography; (ii) clarifying the structural basis of PIWIL4’s R-loop helicase activity (28); (iii) mapping the binding interfaces between PIWIL4 and key partners such as SUPT5H (67), METTL3 (70), and HSP90AA1 (23)—to provide atomic-level insights for rational drug design.

### 7.2 Single-cell heterogeneity and dynamic regulation

PIWIL4 expression varies across tumour tissue. Single-cell sequencing could help clarify: (i) how PIWIL4 expression differs between cancer stem cells and non-stem tumour cells; (ii) what PIWIL4 does in various tumour microenvironment cell types such as CAFs, TAMs, and T cells; and (iii) how PIWIL4 expression changes under therapy and relates to drug resistance acquisition. This should help pinpoint the patient subsets most likely to benefit from PIWIL4-targeted therapy.

### 7.3 PIWIL4 in the tumour immune microenvironment

Several lines of evidence point to PIWIL4’s involvement in anti-tumour immunity. (i) In CRC, the LINC00857/PIWIL4 axis correlates with an immunosuppressive microenvironment (38). (ii) In prostate cancer, PIWIL4 downregulation coincides with NF-κB activation and inflammatory remodelling (62). (iii) In ovarian cancer, PIWIL4 expression links to platinum-based chemotherapy response (58). Yet it is still unclear whether PIWIL4 directly controls immune checkpoint molecules (e.g., PD-L1), chemokines, or T-cell infiltration. Future work should combine immunocompetent mouse models with spatial transcriptomics to systematically map PIWIL4’s functional landscape in the tumour immune microenvironment.

### 7.4 Strategies for developing PIWIL4-specific modulators

Beyond the PROTAC strategy discussed above, several other approaches deserve attention. (i) piRNA mimetics/antagonists: designing oligonucleotides that block PIWIL4–piRNA binding, potentially offering better selectivity. (ii) Allosteric modulators: targeting PIWIL4 conformational intermediates rather than competing at the active site. (iii) AI-driven drug design: using AlphaFold-predicted PIWIL4 structures for virtual screening to speed up lead discovery.

## Glossary

PIWIL4: Piwi-like RNA-mediated gene silencing 4
piRNA: PIWI-interacting RNA
DNMTs: DNA methyltransferases
DNA: Deoxyribonucleic Acid
H3K9me3: Histone H3 lysine 9 trimethylation
LINE-1: Long Interspersed Nuclear Element-1
piR-31115: PIWI-interacting RNA-31115
HSP90AA1: Heat Shock Protein 90 Alpha Family Class A Member 1
*OTX2*: *Orthodenticle Homeobox 2*
AML: acute myeloid leukaemia
HCC: hepatocellular carcinoma
CRC: colorectal cancer
PAZ: Piwi/Argonaute/Zwille
PI3K: Phosphatidylinositol 3-kinase
Akt: Protein Kinase B (PKB)
mTOR: Mammalian target of rapamyci
Wnt: Wingless-related integration site
β-catenin: Beta-catenin
cTNM: Clinical Tumour-Node-Metastasis
IHC: Immunohistochemistry
ER: Estrogen Receptor
EMT: epithelial-mesenchymal transition
p14ARF: p14 Alternative Reading Frame
p53: Tumor Protein p53
*CDKN2A*: Cyclin-Dependent Kinase Inhibitor 2A
p16INK4a: p16 inhibitor of CDK4 family member a
MDM2: Mouse Double Minute 2
MLTC-1: Mouse Leydig Tumor Cell line 1
PDAC: pancreatic ductal adenocarcinoma
TNBC: triple-negative breast cancer
TGF-β: Transforming Growth Factor β
MAPK: Mitogen-Activated Protein Kinase
ERK: Extracellular Signal-Regulated Kinase
FGF: Fibroblast Growth Factor
MHC: Major Histocompatibility Complex
NSCLC: non-small cell lung cancer
MMPs: Matrix Metalloproteinases
CSCs: Cancer stem cells
OCSCs: ovarian cancer stem cells
RFS: recurrence-free survival
RB: Retinoblastoma
CRM: cis-regulatory modules
RBCSCs: Retinoblastoma Cancer Stem Cells
lncRNA: long non-coding RNA
TMB: Tumor Mutational Burden
GDSC: Genomics of Drug Sensitivity in Cancer
CTRP: Cancer Therapeutics Response Portal
CCA: cholangiocarcinoma
piR-31470: PIWI-interacting RNA 31470
GSTP1: Glutathione S-transferase π1
MBD2: Methyl-CpG-binding domain protein 2
RWPE1: Retrovirally Wimmortalized Prostatic Epithelium 1
TLR: Toll-like Receptor
NF-κB: Nuclear factor-κB
ceRNA: competitive endogenous RNA
RNGS: regulatory non-coding RNA-mediated gene silencing
TCGA: The Cancer Genome Atlas
GSE107943: Gene Expression Omnibus
HR: Hazard Ratio
AUC: Area Under the Receiver Operating Characteristic
OS: overall survival
LSCs: leukaemia stem cells
HSPCs: haematopoietic stem and progenitor cells
MYC: v-myc avian myelocytomatosis viral oncogene homolog
ATR: Ataxia Telangiectasia and Rad3-related protein kinase
LDH: Lactate Dehydrogenase
THP-1: Human acute monocytic leukemia cell line THP-1
ROS: Reactive Oxygen Species
RCC: renal cell carcinoma
STS: soft tissue sarcoma
TMB: Tumour Mutational Burden
PAR-CLIP: Photoactivatable Ribonucleoside-Enhanced Crosslinking and Immunoprecipitation
CHAPIR: Cardiac-Hypertrophy-Associated piRNA
METTL3: Methyltransferase-like protein 3
HIV-1: Human Immunodeficiency Virus type 1
SUPT5H: SPT5 homolog, DSIF elongation factor subunit
HP1α: Heterochromatin Protein 1 Alpha
HP1β: Heterochromatin Protein 1 Beta
HP1γ: Heterochromatin Protein 1 Gamma
SETDB1: SET Domain Bifurcated 1
HDAC4: Histone Deacetylase 4
NOA: non-obstructive azoospermia
RPE: Retinal Pigment Epithelium
AMD: age-related macular degeneration
CNV: Choroidal neovascularisation
LINE-1: Long Interspersed Nuclear Element-1
VEGF: Vascular Endothelial Growth Factor
VEGFR2: Vascular Endothelial Growth Factor Receptor 2
*OTC 4*: *Octamer-binding transcription factor 4*
*SOX2*: *SRY-box transcription factor 2*
*β-III tubulin*: *Beta-III tubulin*
MAP2: Microtubule-Associated Protein 2
sALS: sporadic amyotrophic lateral sclerosis
DR: diabetic retinopathy
wAMD: Wet age-related macular degeneration
CHAPIR: hypertrophy-related piRNA
METTL3: Methyltransferase-like protein 3
GDSC: Genomics of Drug Sensitivity in Cancer
CTRP: Cancer Therapeutics Response Portal
PROTAC: Proteolysis-Targeting Chimera
PPI: Protein–Protein Interaction
CAFs: Cancer-Associated Fibroblasts
TAMs: Tumor-Associated Macrophages
PD-L1: Programmed Death-Ligand 1.
